# CircRNA‐associated ceRNA regulatory networks as emerging mechanisms governing the development and biophysiopathology of epilepsy

**DOI:** 10.1111/cns.14735

**Published:** 2024-04-26

**Authors:** Maryam Kohansal, Yasemin Khudiar Alghanimi, Shaimaa R. Banoon, Abdolmajid Ghasemian, Hamed Afkhami, Abdolreza Daraei, Zhangling Wang, Najmeh Nekouian, Jindong Xie, Xinpei Deng, Hailin Tang

**Affiliations:** ^1^ Noncommunicable Diseases Research Center Fasa University of Medical Sciences Fasa Iran; ^2^ Department of Biology Payame Noor University Tehran Iran; ^3^ College of Education for Pure Sciences University of Kerbala Kerbala Iraq; ^4^ Department of Biology, College of Science University of Misan Amarah Iraq; ^5^ Nervous System Stem Cells Research Center Semnan University of Medical Sciences Semnan Iran; ^6^ Cellular and Molecular Research Center Qom University of Medical Sciences Qom Iran; ^7^ Faculty of Medicine Shahed University Tehran Iran; ^8^ Cellular and Molecular Biology Research Center, Health Research Institute Babol University of Medical Sciences Babol Iran; ^9^ State Key Laboratory of Oncology in South China, Guangdong Provincial Clinical Research Center for Cancer Sun Yat‐sen University Cancer Center Guangzhou China

**Keywords:** biogenesis, biomarker, circRNA, circular RNA, competing endogenous RNA, epilepsy, microRNA sponge

## Abstract

The etiology of epilepsy is ascribed to the synchronized aberrant neuronal activity within the brain. Circular RNAs (circRNAs), a class of non‐coding RNAs characterized by their circular structures and covalent linkage, exert a substantial influence on this phenomenon. CircRNAs possess stereotyped replication, transience, repetitiveness, and paroxysm. Additionally, MicroRNA (miRNA) plays a crucial role in the regulation of diverse pathological processes, including epilepsy. CircRNA is of particular significance due to its ability to function as a competing endogenous RNA, thereby sequestering or inhibiting miRNA activity through binding to target mRNA. Our review primarily concentrates on elucidating the pathological and functional roles, as well as the underlying mechanisms, of circRNA–miRNA–mRNA networks in epilepsy. Additionally, it explores the potential utility of these networks for early detection and therapeutic intervention.

## Graphical abstract

CircRNA–miRNA–mRNA networks in epilepsy. CirRNAs can bind to microRNA response elements through sponging and regulate their expression downstream according to the competitive endogenous RNA hypothesis (ceRNA). The circRNA–miRNA–mRNA axes hold potential as valuable tools for comprehending the pathogenesis, diagnosis, and treatment of epilepsy. We summarize the possible regulatory networks of circRNA–miRNA–mRNA in epilepsy.

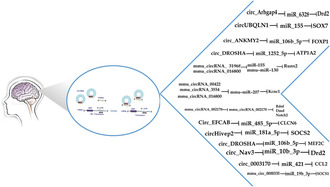

## 1 INTRODUCTION

### 1.1 Epilepsy

Epilepsy encompasses a range of diverse disorders characterized by seizures, which can lead to various neurological consequences such as abnormal neurogenesis, extensive gliosis, inflammation, and neuronal death in the brain.^1^

Epilepsy may afflict individuals of any age, gender, or ethnicity. People of all ages, genders, and ethnicities can be affected by epilepsy. In addition, this condition encompasses various manifestations of seizures and epilepsy syndromes. The etiology of epilepsy involves a range of factors, including hereditary predisposition as well as symptomatic causes such as brain injuries, tumors, infections, strokes, and developmental abnormalities.^2^ Epilepsy is a neurological condition characterized by abnormal nervous transient dysfunction that affects the motor, sensory, consciousness, and autonomic nervous systems and is typically accompanied by abnormal electroencephalograms (EEGs) and pathological abnormalities.^3^ Recurrent seizures can exacerbate cerebral dysfunction, induce neuronal damage and necrosis, and potentially lead to fatal outcomes, imposing a substantial burden on the families of individuals affected by this condition.^4^ Behavioral comorbidities and spontaneous seizures are frequently observed in individuals with epilepsy, a group of chronic neurological disorders characterized by diverse etiologies. A notable proportion of cases fall under the category of acquired epilepsy, despite the fact that the underlying mechanisms of epileptic seizures remain largely unidentified and idiopathic factors are prevalent. The initiation and progression of epileptogenesis, ultimately leading to unprovoked seizures, are frequently associated with prior neurological injuries in this particular form of epilepsy.^5^

### 1.2 Epilepsy and circRNA

**FIGURE 1:**
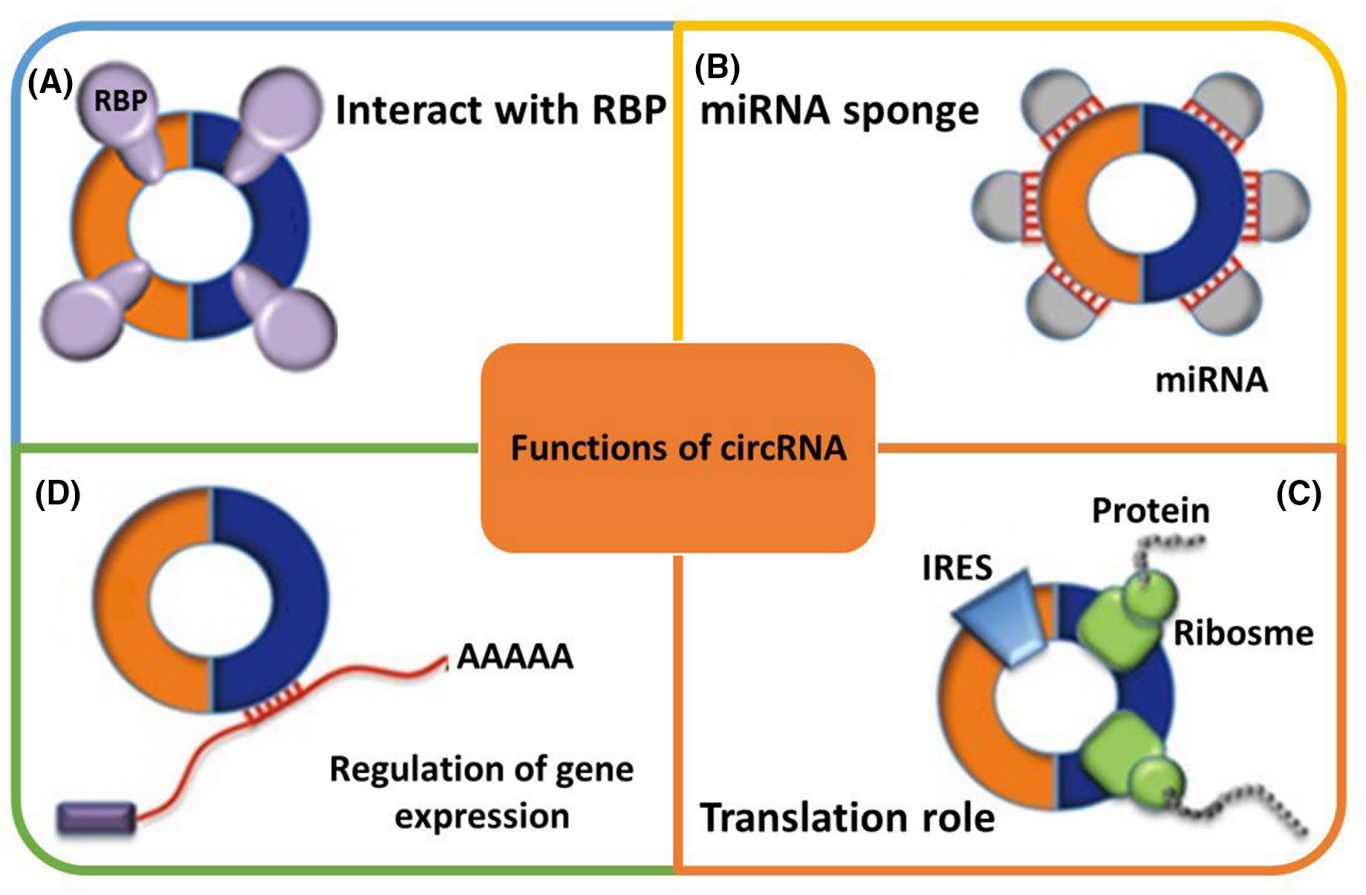
Purpose of circular RNA. (A) Interact with RNA‐binding proteins: When circRNAs bind to RBPs, RNA‐protein complexes might result. (B) Circular RNAs exhibit a miRNA sponge effect by having many miRNA binding sites, which act as ceRNAs when bound to miRNAs. (C) Translation role of circRNA: The eukaryotic ribosomal 40S small subunit can bind to circular RNAs through this location to guide the synthesis of proteins. Circular RNAs have an IRES. (D) Regulation of gene expression; ceRNA, competing endogenous RNA; circRNA, circular RNA; IRES, internal ribosome entry site; miRNA, MicroRNA; RBP, RNA‐binding protein; snRNA, small nuclear RNA.

In the domain of epilepsy, the iterative anomalous firing of neurons leads to the emergence of abrupt, recurring, and fleeting symptoms. A recent survey analysis reveals that an estimated 70 million individuals worldwide are affected by this condition, with 90% of them residing in underdeveloped regions.^6^ The global prevalence of epilepsy ranges from 6.38% to 7.60%.^7^ Annually, there are 67.77 new cases of epilepsy per one hundred thousand people worldwide.^8^ Epilepsy, characterized by recurrent and unpredictable seizures, arises from episodic brain dysfunction. Partial seizures manifest initially in a specific brain region, whereas generalized seizures involve both hemispheres from their onset.^9^ Epileptogenesis involves the manifestation of frequent seizures and subsequent irreversible pathological cerebral changes of heightened magnitude. Except for the importance of controlling seizures clinically, neuron damage caused by recurrent seizures cannot be overlooked. The damage to neurons caused by epilepsy might contribute to recurrent seizures as well.^10^ The etiology of the majority of epilepsy cases remains elusive. Epilepsy can arise from single gene mutations, typically affecting genes responsible for ion channel coding. Temporal lobe epilepsy (TLE), the most prevalent syndrome observed in adults, exhibits a heightened susceptibility to drug resistance. Patients afflicted with TLE often experience brain trauma, infections, status epilepticus, or other brain injuries and are frequently diagnosed with hippocampal pathology, specifically hippocampal sclerosis.^11^ Numerous epigenetic regulatory mechanisms effectively govern the genes implicated in epileptogenesis, and their enduring alteration in the hippocampus may exert a substantial influence on TLE. Among these mechanisms, small noncoding RNAs (ncRNAs) measuring 20–24 base pairs, such as miRNA, have been extensively investigated and are recognized as the most thoroughly studied regulators of gene expression in various diseases.^12^, ^13^, ^14^, ^15^, ^16^, ^17^ Recent research suggests that circRNA modulates gene expression more precisely than other types of ncRNAs.^18^, ^19^ Typically, circRNAs can join forces with RNA‐binding proteins (RBPs) (Figure 1) to form protein complexes that control the transcriptional and parental linear genes' post‐transcriptional expression. It has been suggested that circRNAs serve as sponges for RBPs and scaffolds for protein complexes posttranscriptionally. Precursor miRNAs undergo degradation to generate mature miRNAs, subsequently assembled by the RNA‐induced silencing complex (RISC) comprising the argonaute protein (AGO).^20^ Multiple mechanisms exist through which miRNAs facilitate mRNA degradation or impede mRNA translation by directly or indirectly binding to complementary sequences within the 3′‐untranslated regions (UTRs) of their target mRNAs. Intriguingly, circRNAs possess the capability to engage with miRNAs as competitive endogenous RNA (ceRNA), decoy, or sponge miRNAs, thereby regulating the expression of mRNA. It has also been shown that particular proteins or mRNAs can influence circRNAs through an either negative or positive feedback mechanism by competitively binding to comparable miRNA response elements (MREs).^21^ Despite the relatively low expression of circRNAs compared to miRNAs, in many cases, they are resistant to ribonuclease R, highly conserved, stable, and exhibit development‐stage‐specific and tissue‐specific expression. By lowering miRNA availability, they act as ceRNAs.^22^ Emerging evidence indicates that ceRNAs exert a pivotal influence on cardiovascular diseases, neurological disorders, and cancer. Considering their wide presence in various biological samples, they have the potential to serve as valuable biomarkers for diagnosis or prognosis. Notably, non‐coding RNAs were previously disregarded as mere genomic transcripts in the scientific community approximately one to two decades ago. Nevertheless, they are now understood to be crucial epigenetic regulators of cellular metabolism and survival. Researchers are investigating these minute compounds to identify novel biomarkers for the early detection and treatment of epilepsy.^23^ This paper provides a comprehensive overview of the potential regulatory networks involving circRNA–miRNA–mRNA in the context of epilepsy. It has been demonstrated that ceRNA participates in the proliferation, aging, and differentiation of normal cells, as well as the pathogenesis of tumors and epilepsies. Epilepsy pathogenesis can be affected by RNA transcripts at the transcriptional level through the regulation of ceRNAs.^11^, ^24^ In recent years, an increasing amount of research has elucidated novel functions of the circRNA–miRNA–mRNA axes within the realm of epilepsy. Ongoing studies are shedding light on the distinctive mechanisms underlying both epilepsy and abnormal epilepsy. Consequently, the circRNA–miRNA–mRNA axes present promising prospects as valuable tools for comprehending the pathogenesis, diagnosis, and treatment of epilepsy.

## 2 CHARACTERISTICS

Recent research has revealed and assessed the pivotal role of circRNAs in the regulation of gene expression.^25^ Provicly, these covalently closed circular RNA molecules have been categorized as viroids,^26^ infectious Hepatitis delta virus molecules,^27^ and products of splicing errors.^28^ The expression of these molecules is widespread in human cells, sometimes exceeding 10 times that of their linear isomers.^29^ Linear RNAs exhibit greater stability within the human body as they are less susceptible to digestion by RNase R.^30^ CircRNAs possess a diminutive response element that engages in interactions with miRNAs to govern the expression of target genes. The majority of circRNAs are non‐coding, highly conserved, and predominantly located in the cytoplasm, with only a limited number found in the nucleus.^31^ While a minority of circRNAs participate in transcription, the majority are involved in the regulation of transcription and post‐transcriptional processes.^32^

## 3 THE BIOGENESIS OF circRNAs

circRNAs are predominantly generated through the backsplicing of intronic or exonic sequences within primary transcripts, thereby interfering with the conventional splicing of mRNA. There are three major types of circRNA, based on the diversity of source sequences: intron circRNAs and exonic circRNAs (EcRNAs), as well as intron circRNAs (intron circRNAs), which include tRNA intronic circular RNAs (tricRNAs) and circular intronic RNAs (ciRNAs) from pre‐mRNAs.^18^ The prevailing belief is that the spliceosome machinery in most circRNAs will join a 5‐splice site (donor) of an exon with a 3′‐splice site (acceptor), thereby forming a closed‐loop structure with a specific junction.^33^ A complex network of cis‐acting elements and trans‐acting factors controls this process. Specifically, during circularization, cis‐acting elements located in upstream introns establish complementary base pairing with downstream introns.^34^ RBPs, known for their capacity to act as trans‐acting activators or inhibitors of the circRNA biogenesis machinery, exert a crucial influence on the regulation of circRNA production, as evidenced by previous studies.^20^ Notably, muscleblind (MBL/MBNL1) and the quaking (QKI) protein possess the capability to bind to certain sequence motifs in neighboring introns, linking them together to promote cycling and subsequent circRNA synthesis.

## 4 FUNCTIONS IN EPILEPSY

### 4.1 miRNA sponge

**FIGURE 2:**
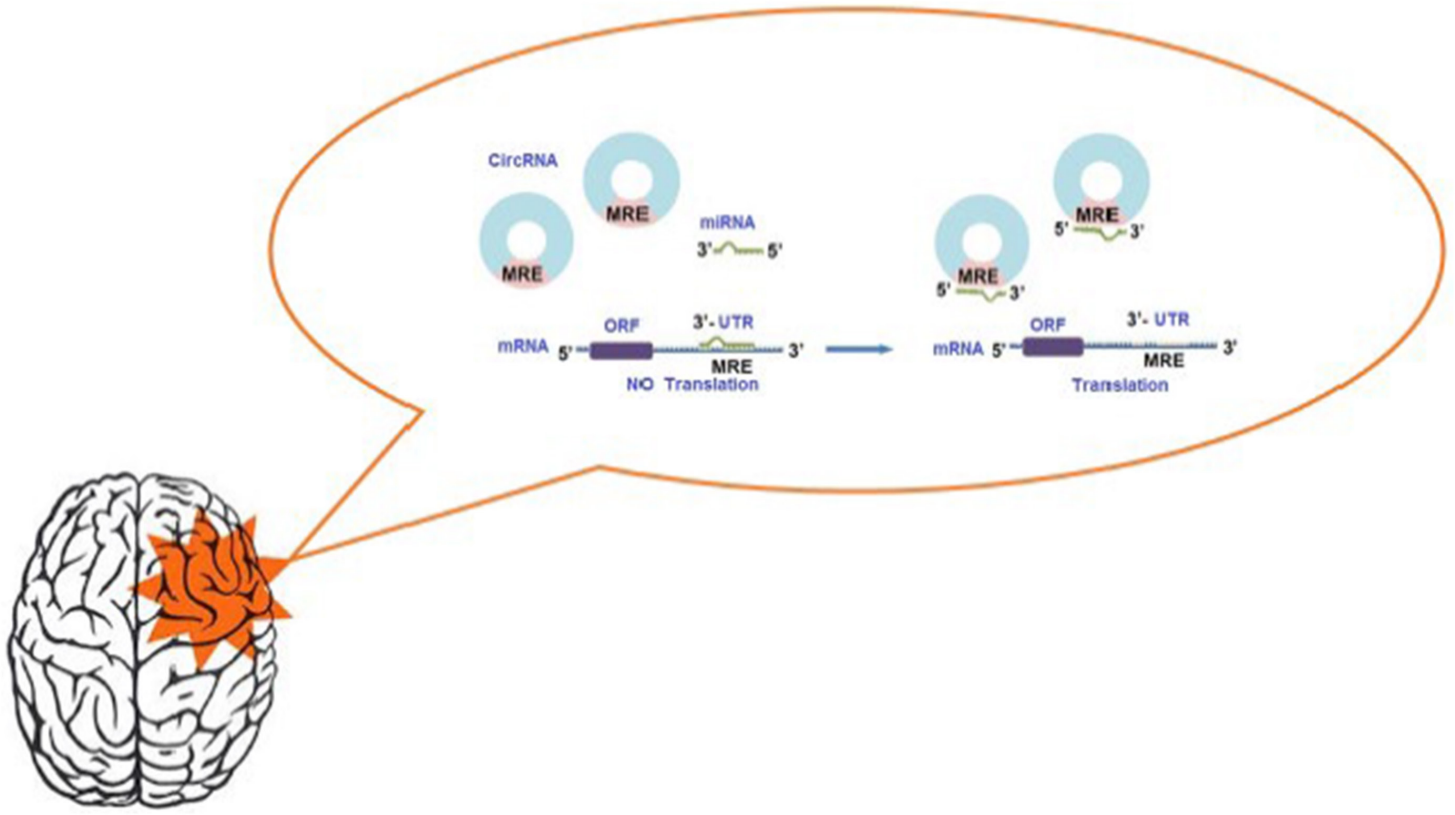
Circular RNAs may have an impact on miRNA activity.

miRNAs are crucial regulators of gene expression by selectively binding to specific regions of mRNA, thereby either facilitating or inhibiting the process of translation.^20^ Emerging evidence suggests that circRNAs can function as miRNA sponges or ceRNAs to impede the action of miRNA, according to growing data^35^ (Figure 2). As sponges for miRNA, circRNAs may have a role in the transcriptional regulation of target genes in malignancies, according to an increasing body of evidence.^18^, ^36^, ^37^, ^38^ CDR1as, also referred to as the antisense transcript to protein 1 associated with cerebellar degeneration, is the initial circRNA that functions as a ceRNA. CDR1as contains over 60 conserved miR‐7 binding sites.^18^ Similar to the knockdown of miR‐7, it inhibited zebrafish midbrain growth and bound to miR‐7.^39^ There have been reports that tangerines, tomatoes, rice, arabidopsis, and wheat contain miRNA sponge functions.^32^

## 5 COMPETITIVE ENDOGENOUS RNA (ceRNA)

CirRNAs can bind to microRNA response elements through sponging and regulate their expression downstream according to the competitive ceRNA.^18^ Consequently, the ceRNA hypothesis emerges as a noteworthy regulatory determinant in the pathological progression of various diseases.

## 6 ceRNA NETWORK IN EPILEPSY

### 6.1 Animal model and cell model studies

Chen et al.^11^ utilized epileptic cell models to investigate the impact of circ_0003170 on hippocampal neuron injuries through the regulation of miR‐421/CCL2. In contrast to miR‐421, Circ_0003170 and C‐C motif chemokine ligand 2 (CCL2) expressions were increased in magnesium‐free (Mg^2+^‐free)‐induced neurons and TLE serum specimens. Circ_0003170 knockdown reduced the cell cycle arrest, oxidative stress, and death that Mg^2+^‐free caused in neurons via enhancing miR‐421. In addition, the present study revealed that the overexpression of miR‐421 led to amelioration of Mg^2+^‐free‐induced cell injury by decreasing CCL2 levels. The suppression of circ_0003170 caused human hippocampus neuron damage caused by Mg^2+^‐free to be lessened by the miR‐421/CCL2 axis. The reversal of the circ_0003170 knockdown was observed upon the overexpression of CCL2.

Notably, Lee et al. conducted an investigation using a mouse epilepsy model to assess the impact of modified circRNA expressions on their respective target miRNAs and mRNAs. The researchers postulated that circRNAs may have a potential role in the pathophysiology of chronic epilepsy. The authors compared the circRNA expression profile in the hippocampus of mice subjected to pilocarpine treatment with that of mice in the control group. Furthermore, they examined the association between the target miRNAs and the miRNA response elements (MREs) present in dysregulated circRNAs. An additional inquiry was undertaken to examine a regulatory network comprising dysregulated circRNAs, miRNAs, and mRNAs that reciprocally target one another, as miRNAs inhibit their target mRNAs. The examination of the identified networks was conducted through the utilization of bioinformatics. As a result, dysregulation of 43 circRNAs in the hippocampal nucleus was observed, with 17 being downregulated and 26 being upregulated. Confirming the notion that circRNAs impact miRNA targets, the expression of MRE in these circRNAs exhibited a negative correlation with the expression of the target miRNA (*r* = −0.461, *p* < 0.001). The analysis of gene ontology detected 333 disrupted circRNA–miRNA–mRNA networks, and further examination of pathways indicated a significant association between the upregulated expression of mRNAs within these networks and the underlying mechanisms of epilepsy.

Among these, STRING analysis identified 37 crucial mRNAs that frequently interacted with other target mRNAs that were dysregulated. Dysregulation of the distinct associations between circRNAs and significant mRNAs was confirmed through the utilization of PCR. STRING analyses revealed that 37 CMI‐mRNAs encoded proteins that displayed robust interconnections, suggesting their potential implication in chronic epilepsy. Moreover, several CMI‐mRNAs were controlled by numerous circRNAs, while others were controlled by a single circRNA, suggesting that these molecules may be important molecules.^40^ For instance, the Kcnc1 gene, which exhibits lower expression levels, encodes the subunit KV3.1 of the voltage‐gated potassium channel and interacts with six additional CMI‐mRNAs identified via STING analysis. Spontaneous seizures occur due to a high‐frequency neuronal firing of inhibitory GABAergic interneurons caused by a loss‐of‐function mutation in the gene.^40^ The regulation of Kcnc1 is influenced by the downregulated 004229 016800, _circRNA_35542, and circRNAs mmu, while it is upregulated by mmu–miR–207. The hippocampus has high levels of expression of Runx2, which is a transcription factor involved in both neuronal excitability mediated by glutamate and cellular homeostasis.^41^ The downregulation of mmu_circRNA_31968 and the upregulation of mmu‐miR‐23a have been found to target Runx2. Additionally, the highly regulated miRNA, mmu_circRNA_002170, has been observed to inhibit the downregulated mmu‐miR‐468 by interacting with increased levels of Notch2, Dmd, and Bdnf. Bdnf, which is encoded by the brain‐derived natriuretic factor (Bdnf) gene, is a neurotrophin known to promote TrkB‐mediated dysregulation, synaptic formation, and abnormal neurogenesis. In the hippocampus and other brain regions, DMDF encodes the protein dystrophin, which keeps kainate‐type glutamate receptors active and plays a role in seizure susceptibility.^42^ Additionally, Notch2 is responsible for abnormal remodeling of neuronal circuits.^43^ The authors of the study have postulated the hypothesis that imbalanced circRNAs may contribute to the pathophysiology of long‐lasting epilepsy by regulating interactions between circRNA−miRNA−mRNA.^24^

In another study, Gomes‐Duarte et al. conducted an investigation utilizing RNA‐sequencing (RNA‐seq) on hippocampal tissue from a rat model of mesial temporal lobe epilepsy (mTLE) induced by perforant pathway stimulation (PPS) to explore the potential role of circRNAs in epilepsy. In this study, a total of 218 circRNAs that were differentially expressed (DE) were identified. It is interesting to note that most of these circRNAs were altered during the first spontaneous seizure (DOFS). Additionally, it was demonstrated that the expression patterns of circ_Arhgap4 and circ_Nav3 were linked to the regulation of miR‐6328 and miR‐10b‐3p, respectively, in terms of their target genes. Bioinformatics analysis was employed to explore the possible association of circ_Arhgap4 and circ_Nav3, along with their predicted miRNA targets, in mTLE. This analysis aimed to identify potential targets of miR‐6328 and miR‐10b‐3p, followed by network analysis of the circRNA/miRNA/mRNA axis. The functional enrichment study revealed that the targets for miR‐10b‐3p and miR‐6328 were obtained through Gene Ontology (GO) terms specifically related to “signaling,” “regulation of biological processes,” and “response to stimulus.” This finding suggests that the observed changes may be attributed to a response in neuronal activity due to circRNA deregulation. This aligns with previous research indicating that circRNA expression is influenced by neuronal activity and synaptic enrichment and supports the higher circRNA expression observed at the DOFS.^44^ Drd2 was significantly upregulated at the DOFS. It is postulated that miR‐6328, a miRNA associated with circ Arhgap4, may regulate the expression of the dopamine receptor D2 (D2R) encoded by Drd2.^45^, ^46^ Drd2 has been associated with neuroactive ligand‐receptor interactions, glutamate receptor binding, signaling events, and dopaminergic synapses through the analysis of GO Function, Reactome, and Kyoto Encyclopedia of Genes and Genomes (KEGG) pathways. Research on mice indicates that there is a correlation between D2R surface expression in the DG and synaptic plasticity as well as memory formation. It is worth noting that these functions are known to be negatively affected in epilepsy.^47^ Extensive investigation has been carried out on the role of D2 receptors in epilepsy, encompassing both animal and human studies.^48^ The dysregulation of D2R has been associated with multiple mechanisms, including increased vulnerability to seizures induced by KA and inhibition of glutamatergic neurotransmission, as well as the modulation of excitotoxicity. Previous research indicates that Drd2 mice exhibit a decreased susceptibility to seizures and increased neurotoxicity when exposed to both pilocarpine and kainic acid, in contrast to wild‐type mice.^46^ Based on this data, it can be inferred that D2R exerts a neuroprotective impact by reducing seizure activity and inducing significant neuronal loss in the hippocampus.^47^ The observed increase in Drd2 expression during the early stages of epilepsy could potentially serve as a protective mechanism against neurodegeneration caused by PPS. Overall, these findings provide support for the hypothesis that the upregulation of circ_Arhgap4 leads to diminished levels of miR‐6328 and heightened D2R expression, potentially offering a defense mechanism against epilepsy‐associated alterations. Several of the circRNAs discovered in this study possess only one or a small number of anticipated miRNA binding sites. Cdr1as, which has over 70 miR‐7 binding sites, is the sole circRNA with numerous miRNA binding sites.^49^ Currently, it is suggested that circRNA and miRNA abundance should be similar in order for sponges to function effectively.^50^ To enhance the quantity of circRNA duplicates, one may utilize numerous binding sites for a specific miRNA, thereby facilitating this process.^51^ In cellular sections like synapses, certain subsets of circRNAs are highly enriched. Aside from miRNA sponging, it is plausible that the circ_Arhgap4/circ_Nav3 axis modulates Drd2 and other targets through binding to RBPs or controlling transcription. An intriguing connection exists between the dysregulation of circRNA and the transformation of brain networks into a state of spontaneous seizure activity. Based on these findings, it can gain a better understanding of circRNA function in TLE by using a molecular framework.^34^

As part of a severe epilepsy model in mice, Liao et al. screened the DE circRNAs, along with an analysis of the associated microRNAs and messenger RNAs, to explore the pathways implicated in their enrichment and functionality. NGS technology was applied by researchers to analyze the circRNA expression profiles of the genomes of mice with acute epilepsy (*n* = 6) compared to mice with normal gene expression. The researchers identified multiple circRNAs, with 66 showing downregulation and 34 showing upregulation. Key genes mm9_circ_004424 and mm9_circ_008777 were detected using quantitative PCR (qPCR) in the epilepsy group, where they exhibited high expression levels. Subsequently, a regulatory network involving mm9_circ_008777 and mm9_circ_004424 was determined to be crucial for the circRNA–miRNA–mRNA interaction. To ascertain the involvement of Bisphenol S (BPS) and signaling pathways in the regulation of differential gene expression, KEGG pathway and GO function analyses were employed. The authors identified pathways related to metabolic processes, biological regulation, binding, catalytic activity, and cellular processes, as well as VEGF, ErbB, GnRH, chemokine, and T‐cell receptor signaling pathways. In this way, they found that mm9_circ_008777 and mm9_circ_004424 may carry out GO functions via biological regulation, metabolic processes, catalytic activity, and cellular processes, and may contribute to epilepsy through signaling pathways such as VEGF, GnRH, chemokine, and ErbB. The identification of MM9_circ_004424 and MM9_circ_008777 as potential biomarkers for epilepsy diagnosis suggests that clinical interventions targeting these circRNAs could be beneficial in the management of epilepsy.^52^

In a separate investigation, Zhu et al. reported that CircRNA Ubiquilin1 (circUBQLN1) functions as a competitive endogenous RNA for miR‐155, forming a complex with miR‐155 to modulate the expression of sex‐determining region Y‐box 7 (SOX7). The co‐regulation of circUBQLN1 and miR‐155 is involved in the regulation of SOX7 expression. Overexpression of circUBQLN1 mitigated Mg^2+^‐free‐induced nerve injury, and the protective effects of circUBQLN1 and miR‐155 in human neurons‐hippocampal (HN‐h) cells exposed to Mg^2+^‐free conditions were reversed upon knockdown of SOX7. A prior investigation identified circUBQLN1 as being downregulated in epilepsy samples and located at chr9: 86274695–86284242.^20^, ^52^ The discovery of Y‐box 7 (SOX7), a sex‐determining region in epilepsy's hippocampal neurons, revealed its ability to suppress cell death.^24^ Based on this data, circUBQLN1 lacked the 3′ poly(A) tail, which prevented its binding to oligo (dT) 18 primers during reverse transcription. After conducting an analysis on the stability of RNase R, it was found that circUBQLN1 exhibited significantly higher stability in comparison to linear RNA. Generally, exonic circRNAs are localized in the cytoplasm. Moreover, HN‐h cells containing circUBQLN1, when subjected to Mg^2+^‐free solutions, exhibited cytoplasmic localization. In HN‐h cells treated with Mg^2+^‐free, circUBQLN1's cytoplasmic localization was confirmed consistently, and circUBQLN1 was also expressed abnormally both in human epilepsy samples and in the Mg^2+^‐free‐induced cell model. Subsequent to this, circUBQLN1 function in epilepsy was analyzed in vitro. The findings of this investigation indicated that circUBQLN1 overexpression reduced Mg^2+^‐free‐induced neurological damage, including proliferation inhibition, oxidative response, and apoptosis promotion, in HN‐h cells. It appears that circUBQLN1 may inhibit epilepsy in vitro. MiR‐155 was identified as a potential miRNA target in this study for circUBQLN1. The researchers anticipated and confirmed that circUBQLN1 acts as a sponge for miR‐155. In addition, the 3′UTR of SOX7 contained binding sites for miR‐155, which directly downregulated SOX7 expression. The protective role of circUBQLN1 was associated with sponging miR‐155, as miR‐155 overexpression inhibited the effects of circUBQLN1 against Mg^2+^‐free‐induced cell damage. Silence of SOX7 also prevented inhibition of Mg^2+^‐free induced epilepsy by miR‐155 inhibitors and circUBQLN1 overexpression. So circUBQLN1 may exert a positive effect on SOX7 levels by interacting with miR‐155. Validation of the circUBQLN1/miR‐155/SOX7 axis was conducted in epilepsy. The results of these comprehensive studies showed that circUBQLN1 suppressed epileptogenesis and increased SOX7 expression as a miR‐155 sponge. A signal network involving circUBQLN1/miR‐155/SOX7 helped shed light on epilepsy's pathological mechanism. It could be beneficial to use circUBQLN1 as a therapeutic target to treat epilepsy. CircUBQLN1 regulates the miR‐155/SOX7 axis to inhibit nerve injury in HN‐h cells treated without Mg^2+^. For the treatment of epilepsy, cirUBQLN1 may offer great therapeutic potential.^9^

In another investigation, Xiaoying et al. suggest that circHivep2 regulates microglia activation in the progression of epilepsy by interfering with miR‐181a‐5p to promote suppressor of cytokine signaling 2 (SOCS2) expression.^53^ A total of 1519 circRNAs were detected in the profile, with 892 demonstrating a decrease in expression and 627 displaying an increase in expression. Out of these various circRNAs with differential expression, circHiveP2 was specifically chosen by the researchers for further examination because it consistently showed a decrease in animal models of kainic acid (KA)‐induced epileptic seizures. This choice was motivated by the association of HIVEP2 with various regulatory pathways impacting cellular immunity, developmental processes, and neurological disorders in humans. The researchers discovered via an online study that miR‐181a‐5p and circHivep2 might interact online, and a dual‐luciferase reporter experiment verified this. Through the stimulation of the STAT3 pathway, miR181a‐5p collaborates with fibroblast growth factor receptor 3 (FGFR3) to advance bladder cancer. It is interesting that epilepsy has been linked to both FGFR3 and STAT3. Inhibition of STAT3 was observed to reduce seizures in pilocarpine‐induced SE, while FGFR3 mutations have been linked to focal epilepsy and bilateral medial temporal lobe abnormalities. The researchers discovered that Cytokine Signaling 2 is a target of miR‐181a‐5p as well. In the past, epilepsy has been linked to SOCS proteins, which may provide a contraindication to cytokine interaction. In another study utilizing a pentylenetetrazole‐induced rat model of epilepsy, Song et al. observed a significant upregulation of Toll‐like receptor (TLR) 4 and TLR2.^54^ This upregulation led to the suppression of Cytokine Signaling 3 and SOCS1 expression, ultimately resulting in the upregulation of STAT3 expression. Type I interferon (IFN) signaling can be inhibited by SOCS proteins, which also negatively influence TLR‐mediated immunological responses. In the study, IL‐1β, TNF‐α, and Iba‐1 levels were found to be high in KA‐induced epilepsy, whereas overexpression of circHivep2 dramatically decreased the elevated levels of IL‐1β and TNF‐α. The highest levels of IL‐1β and TNF‐α were produced by the underexpression of circHivep2. Contrarily, it was found that upregulation of miR181a‐5p counteracted the inhibitory impact of circHivep2 on the activation of microglial cells. These findings imply that miR‐181a‐5p is negatively regulated by circHivep2 to inhibit the upregulation of IL‐1β, TNF‐α, and microglia cell activation. Additionally, they discovered that upregulation of miR‐181a‐5p suppresses the expression of SOCS2, leading to the activation of pro‐inflammatory signaling pathways. The expression of miR‐181a‐5p was observed following exposure to KA, but this was hindered in the presence of circHivep2. It revealed a potential interaction between circHivep2 and miR‐181a‐5p, possibly involving binding or regulation of expression. The researchers' ultimate objective was to investigate the potential anticonvulsant effects of circHivep2 in a mouse model of epileptic seizures induced by KA. Upon administration of circHivep2+ exosome treatment to mice with KA‐induced epilepsy, a significant suppression of microglial activation was observed. At 72 h following KA‐induced epilepsy, circHivep2+ exosomes further reduced the levels of pro‐inflammatory cytokines, IL‐1β, cytokine release, and TNF‐α in the hippocampus. These results imply that circHivep2 may function as a component of the pathway circHivep2/miRNA‐181a‐5p/SOCS2, which may be useful in controlling epileptic episodes. In summary, this research discovered that circHivep2 could reduce neuroinflammation and the reduction of epileptic seizures' symptoms in a mouse model by activating microglial cells through the inhibition of miRNA‐181a‐5p/SOCS2. CircHivep2 may be employed as a treatment method to delay the onset of epilepsy.^53^

In another investigation, Zhu et al. discovered that mmu_circ_0000335 overexpression drove microglia and macrophages toward M2 polarization, while epilepsy may modify them toward M1 polarization. These results demonstrated that 364 circRNAs were differently expressed between control and epilepsy tissues. Particularly, the expression of mmu circ 0000335 was markedly downregulated in epileptic mice. Overexpression of mmu_circ_0000335 accelerated the induction of the M2 macrophage phenotype in BV2 cells by increasing the expression of Ym1, CD206, IL10, and Arg1. Conversely, the expressions of IL‐6, IL‐1, IFN, and markers of M1 macrophages were reduced in epileptic conditions. The luciferase reporter assay revealed that the expression of mmu_circ_0000335 led to the upregulation of the suppressor of SOCS1 by reducing miR‐19b‐3p levels. A STAT‐binding site may be found in the SOCS1 promoter region, which is situated at 16p13.^55^ Cytokine Signaling 1 is a member of the SOCS family. Numerous cytokines control the expression of SOCS1 in various tissues.^56^ A suppressive effect on inflammation has been discovered for SOCS1 in previous research.^57^ Mmu_circ_0000335 promoted SOCS1 expression, according to the findings of the present investigation, which under epileptic circumstances changed the balance between microglia and macrophages in favor of M2 polarization. The current study's findings showed that mmu_circ_0000335 promoted SOCS1 expression.^58^ Additionally, findings from in vivo studies indicated that the upregulation of mmu_circ_0000335 shifted the equilibrium of macrophages and microglia toward M2 polarization in the context of epilepsy. This resulted in a mitigation of epilepsy‐induced neuronal damage in the hippocampus, as well as a reduction in the production of inflammatory factors mediated by microglia. Ultimately, the authors established that mmu_circ_0000335 attenuated M1 microglia‐induced apoptosis of hippocampal neurons by modulating the miR‐19b‐3p/SOCS1 axis in a murine model of epilepsy.

### 6.2 Human studies

Gong et al. conducted a study examining 586 DE circRNAs in control tissues and tissues from patients with TLE. The findings revealed a significant downregulation of circRNA‐0067835 expression in TLE patients' tissues and plasma. Increased seizure frequency, high HS, and higher Engel scores were associated with lower circRNA‐0067835. It has been suggested that drug‐resistant TLE is related to the downregulation of circRNA‐0067835. In the plasma and tissues of 22 patients with TLE, circRNA‐0067835 was found to be decreased. Bioinformatics analysis suggested that circRNA‐0067835 functions as a ceRNA by sequestering miR‐155 and thereby modulating the expression of FOXO3a. Subsequent validation through luciferase reporter assays confirmed the regulatory role of miR‐155 and CircRNA‐0067835 in modulating the downstream signaling pathways. The authors also attempted to identify the immediate downstream signaling pathways modulated by miR‐155 and circRNA‐0067835. Through bioinformatics analysis and luciferase reporter assays, the FOXO3a gene was identified as a direct target of miR155. The downregulation of FOXO3a mRNA expression in TLE tissues was found to have a negative correlation with miR‐155 expression and a positive correlation with HOXA‐AS2 expression. Through a luciferase test, it was demonstrated that circRNA‐0067835 and FOXO3a interact specifically due to competition for miR‐155. By acting as a sponge for miR‐155 and promoting FOXO3a expression, circRNA‐0067835 was shown to regulate the progression of refractory epilepsy. This suggests that circRNA‐0067835 may serve as a potential therapeutic target for patients with TLE.^59^

Lin et al. demonstrated that the miR‐106b‐5p/FOXP1 regulatory pathway is modulated by circ ANKMY2, impacting the progression of TLE. The findings suggest circ ANKMY2 as a promising therapeutic target for TLE. Elevated levels of miR‐106b‐5p were observed in TLE tissues, while circ ANKMY2 and Forkhead Box Protein 1 (FOXP1) were downregulated. In a rodent TLE model, overexpressing circ ANKMY2 decreased the occurrence of spontaneous recurring seizures (SRSs) and hindered colony formation, suppressed cell growth, and triggered cell apoptosis in cells of the SK‐N‐AS line. Validating the regulatory function of circ ANKMY2 as a competitive endogenous RNA for miR‐106b‐5p holds significant importance. MiR‐106b‐5p mimics also eliminated the impact of increased circ ANKMY2 levels on the formation of colonies, cell proliferation, and apoptosis in SK‐N‐AS cells. FOXP1 was also targeted by miR‐106b‐5p. The silence of FOXP1 also reversed the impact of miR‐106b‐5p inhibitors on the growth, formation of colonies, and apoptosis of SK‐N‐AS cells. In colorectal cancer, it has been shown that miR‐106b‐5p functions as a suppressor of tumor growth.^60^ In addition, miR‐106b knockdown reduced inflammatory and joint damage reactions in collagen‐induced arthritis.^61^ An et al. stated that the correlation between serum miR106b and NHS3 scores existed among individuals with epilepsy.^62^ Additionally, the presence of miR‐106b in the bloodstream could serve as a valuable clinical predictor and a potential diagnostic indicator for childhood epilepsy. These findings showed that miR‐106b‐5p was a detrimental factor in TLE, while circ ANKMY2 governed the advancement of this condition by controlling the activity of miR‐106b‐5p. The authors also found that miR‐106b‐5p targeted the FOXP1 genes in a subsequent manner. Feng et al. showed that the reduction of miR‐183 expression resulted in an increase in FOXP1 expression in rats with epilepsy, leading to a decrease in hippocampus neuron damage.^63^ Additionally, in forebrain pyramidal neurons, FOXP1 controlled the expression of genes necessary for spatial cognition and synaptic flexibility.^64^ Epilepsy and autism spectrum diseases may result from a genetic variation‐related de novo FOXP1 mutation.^65^ The circ ANKMY2/miR106b‐5p axis was found to be able to influence FOXP1 expression in this study. Additionally, the inhibition of FOXP1 restored the impacts of miR‐106b‐5p repression on the growth, formation of colonies, and programmed cell death in SK‐N‐AS cells. Hence, the results indicated that circ ANKMY2 regulated the advancement of TLE by increasing the expression of FOXP1 through miR‐106b‐5p.^57^

As for the TLE cell model and serum samples from TLE patients, Zheng et al. found that circ_DROSHA was downregulated. The upregulation of circRNA Drosha ribonuclease III (circ_DROSHA) alleviated the cytotoxicity of the TLE cell model by enhancing cell proliferation and inhibiting apoptosis. The direct binding of circ_DROSHA to miR‐106b‐5p is significant, as miR‐106b‐5p plays a crucial role in the downstream effector function of circ_DROSHA. Deficits of miR‐106b‐5p relieve inflammation and joint injury in collagen‐induced arthritis mice.^61^ Cerebral ischemia/reperfusion damage can be prevented by suppressing miR‐106b‐5p, which is also increased during acute ischemic stroke, through its interaction with myeloid cell leukemia‐1 (Mcl‐1).^66^ Moreover, miR‐106b‐5p has been identified as the primary biomarker for epilepsy among the dysregulated miRNAs examined.^67^ Circ_DROSHA mediates myocyte‐specific enhancer factor 2C (MEF2C) expression by acting as a miR‐106b‐5p sponge. MEF2C has also been shown to modulate the production of inflammatory mediators and signal transduction pathways. Based on the starBase v.3 database, the authors found that miR‐106b‐5p directly targeted and inhibited MEF2C. Several neuropsychiatric and neurodevelopmental problems have been associated with a neuronal transcription factor.^68^ As an interesting point of interest, MEF2C is also involved in the pathogenesis of epilepsy.^69^ This study reveals that miR‐106b‐5p regulates magnesium‐free solution‐induced cytotoxicity by targeting MEF2C. The findings from this study suggest that circulating circ_DROSHA could serve as a potential biomarker for the clinical diagnosis of temporal lobe epilepsy in a limited sample of patients.^70^

Gray et al. conducted a study comparing alterations in circRNA expression patterns between the hippocampus and temporal cortex of individuals with pharmacoresistant mesial temporal lobe epilepsy (MTLE) and those without the condition. A total of 9 circRNAs (NMD3, FBXL4, TLL1, NCAM1, AC067956.1, BNIP3L, RPS6KC1, HOMER1, and CAMSAP1) displayed significant differential expression. CircRNA‐HOMER1 is also detected in synapses. The bioinformatics workflow showed that out of the nine DE circRNAs, seven of them function as miRNA sponges. There was a shared set of miRNAs between circRNA‐NMD3 and the group of miRNAs that interacted with circRNA‐FBXL4. This observation may be attributed to the conservation of sequences among miRNAs within the same family, as miRNA families frequently exhibit shared sequence, structure, and functionality.^71^ CircNMD3 is generated by the gene locus of the ribosome export adaptor NMD3, which encodes a protein responsible for transporting the cytoplasmic ribosomal 60S subunit.^71^ In MTLE patients' hippocampus, CircNMD3 is demonstrated to be DE. Fifteen miRNAs interact with CircRNA‐NMD3, and additional research on certain miRNAs revealed the DE of their target linear transcripts. In the hippocampus, 16 out of 27 DE transcripts exhibited downregulation, while 11 showed upregulation. The leucine‐rich repeat protein 4 and F‐Box gene loci are where CircRNA‐FBXL4 is expressed. To preserve mtDNA, the FBXL4 protein is colocalized with mitochondria. Among the 57 miRNAs that target CircRNA‐FBXL4, seven belong to the hsa‐miR‐378 family. Of the 83 DE transcripts, 50 showed downregulation, while 33 exhibited upregulation. The authors observed significant differences in the expression levels of circRNA‐HOMER1 and linear transcripts between the MTLE hippocampus and controls. Proteins encoded by the HOMER1 gene engage in neuronal functions by connecting inositol 1,4,5‐triphosphate receptors (IP3Rs) to Group I metabotopic glutamate receptors (mGluR1/5) located on the endoplasmic reticulum, NMDA receptor signaling complexes, and synaptic calcium ion channels. The early gene HOMER1a's brief splice variant, formed following synaptic activity, might hinder the activation of group I mGluR.^72^ Epileptogenesis is connected to HOMER1a.^73^ There are 12 miRNAs that can bind to circRNA‐HOMER1. Gene set enrichment analysis has shown that heterocyclic chemical binding, signal transmission, and transcriptional regulation are involved in the maintenance of the structure and function of hippocampal neurons. Among the 37 transcripts identified, 12 exhibited upregulation, while 25 showed downregulation. The authors further identified miRNA binding sites within the DE circRNA sequences, with changes in complementary miRNA levels being associated with mRNA expression levels. The circRNA–miRNA–mRNA interaction networks in MTLE provide insights into the underlying molecular changes, which could potentially be pathogenic or attributed to treatments, medical interventions, or the disease itself. Based on these results, DE circRNAs and their associated miRNAs may serve as promising therapeutic targets.^74^

Li et al. reported that dysregulations in circRNAs could be indicative of the pathophysiology of TLE.^75^ Circ‐DROSHA and circ‐EFCAB2 expression levels were clearly altered in the temporal cortex tissue of TLE patients, suggesting that circ‐EFCAB2 and circ‐DROSHA may be potential treatment targets and biomarkers in TLE patients. Circ‐DROSHA and circ‐EFCAB2, derived from exons, may regulate host genes. A total of 78,983 circRNAs were identified, with 84.71% being new and 15.29% previously known. Among these, 442 circRNAs showed differential expression between non‐temporal lobe epilepsy and TLE groups, with 254 downregulated and 188 upregulated in TLE patients. Eight circRNAs were validated by real‐time PCR. Surprisingly, in the TLE group compared to the non‐TLE group, circ‐EFCAB2 was dramatically upregulated while circ‐DROSHA expression was markedly downregulated. Drosharibonuclease III (DROSHA) and EF‐hand calcium‐binding domain‐containing protein 2 (EFCAB2) separately serve as the host genes for circ‐DROSHA and circ‐EFCAB2. The EFCAB gene family mostly binds with calcium ions (Ca^2+^) non‐covalently and selectively.^76^ The chloride channel family (CLCs) includes the miRNA‐targeting gene chloride channel 6 (CLCN6) and is linked to a variety of physiological processes, including electrical excitability, electroneutrality, ionic homeostasis, and transepithelial transport.^77^ Blood samples from individuals with mesial TLE exhibit altered CLCN6 as well. The Circ‐DROSHA‐targeting miRNA network selected miR‐1252‐5p based on its highest score. Its target gene, the alpha 2 subunit of Na^+^, K^+^‐ATPase (ATP1A2), has a close connection to epilepsy.^78^ ATP1A2 plays several important roles, including removing extracellular K^+^ and limiting neuronal depolarization during high neuronal excitability.^79^ Additionally, ATP1A2 has been identified as a potential gene associated with human epilepsy.^51^ In view of the fact that circEFCAB2 and circ‐DROSHA are predicted to act as sponges for these miRNAs, they might absorb these miRNAs, which would then suppress the expression of the target genes and have an impact on the pathogenesis of TLE. These circRNAs could provide new insight into the mechanisms underlying TLE. This research advances our understanding of the processes that control noncoding and coding gene expression in TLE and the modifications brought on when the temporal cortex is subjected to continuous seizure activity.^75^

## 7 CONCLUSIONS AND FUTURE PERSPECTIVES

**FIGURE 3:**
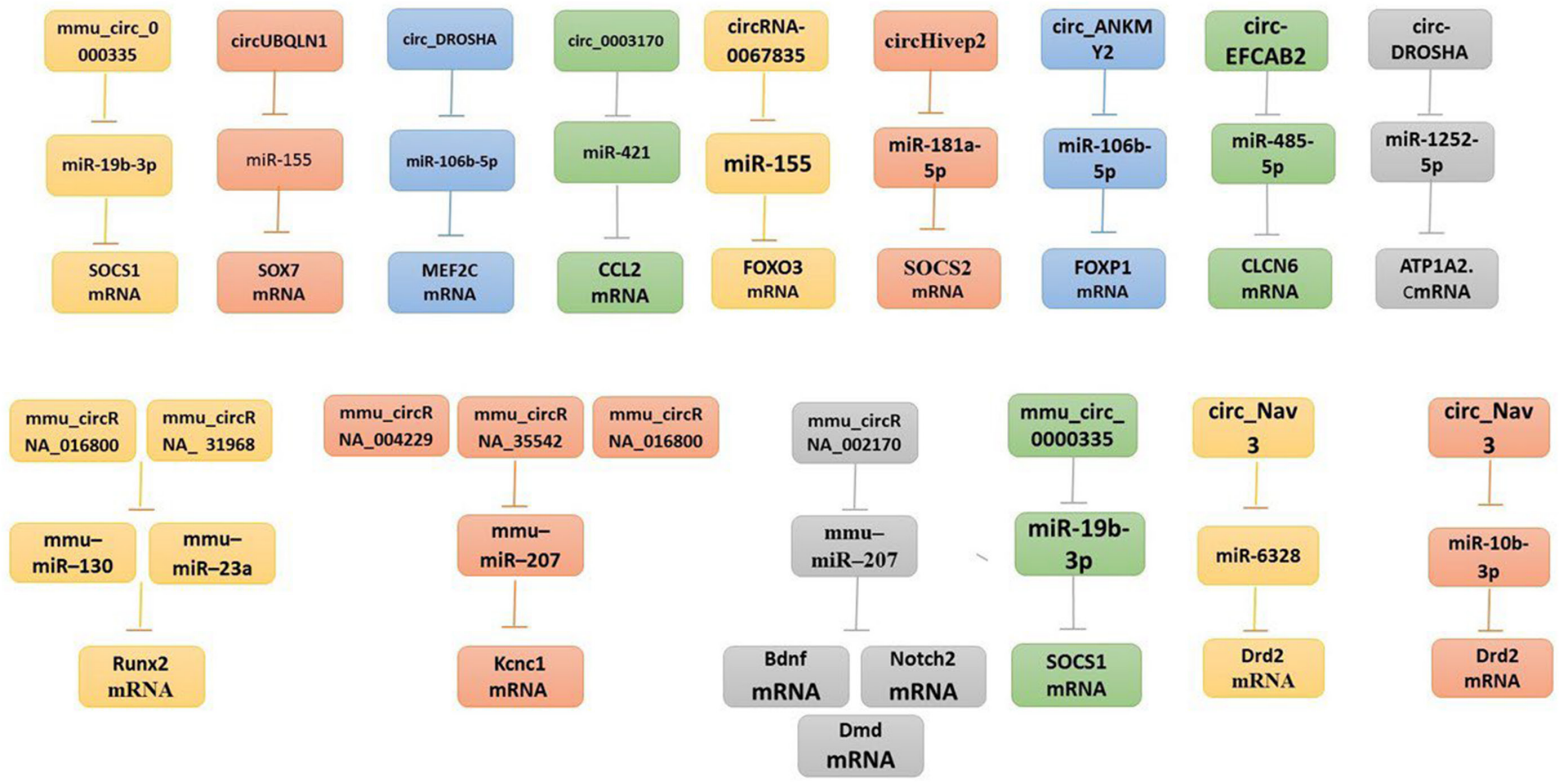
CircRNAs that are dysregulated and functioning circEpilepsy and RNA–miRNA–mRNA.

The expression of circRNA at various stages of experimental epilepsy is profiled for the first time in our study (Figure 3 and Table 1). We demonstrate that the process of epileptogenesis leads to an active regulation of circRNA expression. These findings lend support to the hypothesis that circRNAs may contribute to the pathogenic progression of TLE.

**TABLE 1:** Summary of ceRNA network in epilepsy.

CircRNAs	miRNAs	mRNAs	Expression change (circRNA)	Related molecules, pathways or functions and uses	Sample type	References
circRNA‐0067835	miR‐155	FOXO3	Down	circRNA‐0067835 functioned as a sponge for miR‐155 to control FOXO3a expression.	Temporal lobe epilepsy (Human)	59
mmu_circRNA_31968	mmu‐miR‐23a	Runx2	Down	The MAPK signaling pathway, pathways in cancer, the PI3K‐Akt signaling pathway, focal adhesion, and the Ras signaling pathway	Epilepsy (Animal model)	24
mmu_circRNA_016800	mmu‐miR‐130					
mmu_circRNA_004229	mmu‐miR‐207	Kcnc1	Down			
mmu_circRNA_35542						
mmu_circRNA_016800						
mmu_circRNA_002170	mmu‐miR‐207	Bdnf, Dmd, Notch2	Up			
circ‐EFCAB2	miR‐485‐5p	CLCN6	Down	Stop the expression of the target gene, and affect the pathogenesis of temporal lobe epilepsy	Temporal lobe epilepsy (Human)	75
circ‐DROSHA	miR‐1252‐5p	ATP1A2. C	Up			
circ_ANKMY2	miR‐106b‐5p	FOXP1	Down	Regulated miR‐106b‐5p is used to sponge MEF2C expression	Temporal lobe epilepsy (Human)	80
circHivep2	miR‐181a‐5p	SOCS2	Down	Inhibited microglial activation	Epilepsy (Animal model)	53
mmu_circ_0000335	miR‐19b‐3p	SOCS1	Up	mmu_circ_0000335 overexpression pushed microglia/macrophages toward M2 polarization	Epilepsy (Animal model)	58
circUBQLN1	miR‐155	SOX7	Down	In HN‐h cells treated with Mg^2+^‐free, circUBQLN1 overexpression promoted proliferation but decreased apoptosis and oxidative stress	Epilepsy (cell model)	10
circ_Arhgap4	miR‐6328	Drd2	Up	To compete with mRNAs for miRNA binding via MREs	Mesial temporal lobe epilepsy (Animal model)	23
circ_Nav3	miR‐10b‐3p	Drd2				
circ_DROSHA	miR‐106b‐5p	MEF2C	Down	By enhancing cell proliferation and repressing cell apoptosis, Circ_DROSHA upregulation reduced the cytotoxicity.	Temporal lobe epilepsy (Human)	70
circ_0003170	miR‐421	CCL2	Unknown	Sponging miR‐421	Temporal lobe (cell model)	11

Limited understanding exists regarding the mechanisms involved in the transformation of a post‐insult but pre‐epileptic network into a brain exhibiting recurrent spontaneous seizures. The observed alterations in circRNA expression during this transitional phase are notable, particularly as this enrichment is specific to circRNAs and mRNAs rather than miRNAs.^23^ Changes in circRNA expression may offer an opportunity for intervention to prevent the progression of epilepsy if they play a significant role during the transitional phase.

## AUTHOR CONTRIBUTIONS

All authors participated in the present study. Conceptualization, Hailin Tang, Xinpei Deng, Jindong Xie and Maryam Kohansal; writing—original draft preparation, Maryam Kohansal and Yasemin Khudiar Alghanimi; Data curation, Maryam Kohansal and Shaimaa R. Banoon; Validation, Abdolmajid Ghasemian and Abdolreza Daraei; visualization, Hamed Afkhami, Zhangling Wang and Najmeh Nekouian; writing—review and editing, Xinpei Deng, Jindong Xie and Hailin Tang. All authors have read and agreed to the published version of the manuscript.

## CONFLICT OF INTEREST STATEMENT

The authors declare no conflicts of interest.

## Data Availability

Data sharing is not applicable to this article as no new data were created or analyzed in this study.
